# Buried Interface Passivation Using Organic Ammonium Salts for Efficient Inverted CsMAFA Perovskite Solar Cell Performance

**DOI:** 10.1021/acsomega.4c02656

**Published:** 2024-05-16

**Authors:** Ching-Ho Tien, Wei-Shuo Lai, Lung-Chien Chen

**Affiliations:** † Department of Electronic Engineering, Ming Chi University of Technology, No. 84, Gungjuan Rd., New Taipei City 24301, Taiwan; ‡ Organic Electronics Research Center, Ming Chi University of Technology, No. 84, Gungjuan Rd., New Taipei City 24301, Taiwan; § Department of Electro-Optical Engineering, National Taipei University of Technology, No. 1, Sec. 3, Chung-Hsiao E. Rd., Taipei 10608, Taiwan

## Abstract

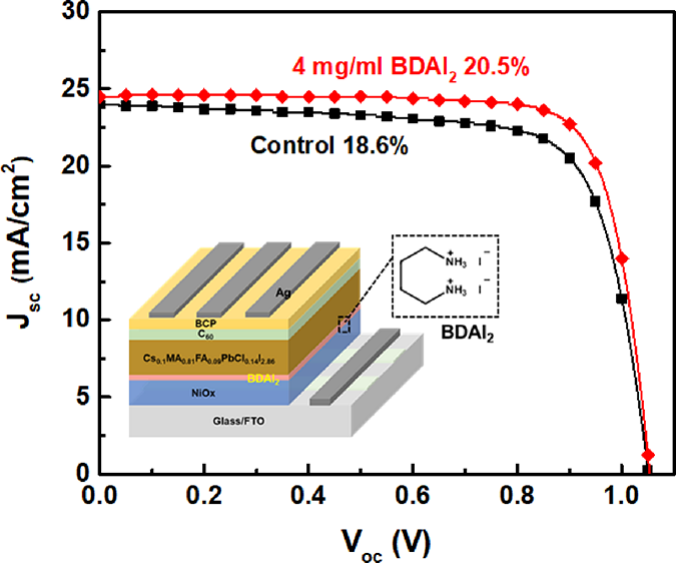

This study uses different doping ratios of CsCl and MACl dual additives to improve the quality of the perovskite, where CsCl reduces the perovskite trap density and increases the resistance of charge recombination, and MACl was used to improve the phase stability. Finally, the composition of Cs_0.1_MA_0.09_FA_0.81_PbCl_0.14_I_2.86_ perovskite solar cell (PeSC) can achieve better open-circuit voltage (Voc), short-circuit current density (Jsc), and photoelectric conversion efficiency (PCE). To achieve a better PCE of PeSC, the use of organic ammonium salt butane-1,4-diammonium iodide (BDAI_2_) to passivate the perovskite bottom surface (buried interface) can effectively suppress the formation of defects at the perovskite buried interface, obtain higher crystallinity, and thereby reduce the probability of carrier recombination. The Jsc, fill factor (FF), and PCE of the PeSC based on BDAI_2_ passivation increased from 24.0 mA cm^–2^, 74.1%, and 18.6% to 24.5 mA cm^–2^, 79.9%, and 20.5%, respectively.

## 1 Introduction

Perovskite solar cells (PeSCs) were an emerging photovoltaic technology that have many advantages, such as high photoelectric conversion efficiency (PCE) (reaching 26.1% in laboratory tests),^1^ suitability for large-scale production, low cost, etc. These features make PeSCs promising to replace traditional silicon solar cells in the future and become the next generation of photovoltaic products.^2−9^ From the perspective of practical applications, the efficiency of PeSCs has reached industrialization requirements; therefore, the stability of the device has become one of the key factors restricting its commercialization. Research shows that ternary-cation perovskites mixed with cesium ions (Cs^+^), formamidinium ions (HC(NH_2_)^2+^, FA^+^), and methylamine ions (CH_3_NH_3_^+^, MA^+^) have attracted widespread attention due to their excellent stability and photoelectric properties,^10−12^ for example, Cs_0.05_(FA_0.83_MA_0.17_)_0.95_Pb(I_0.83_Br_0.17_)_3_ was currently one of the key research component systems for high-efficiency and high-stable PeSCs.^13−15^

The structure of PeSC was generally transparent conductive glass/hole transport layer/perovskite absorber layer/electron transport layer/metal electrode (inverted structure as an example);^16^ during the deposition process of perovskite films, defects were easily generated at the interface between the electron/hole transport layer and perovskite absorber layer, which have adverse effects on the performance of PeSCs.^17,18^

The defects at the interface of the perovskite light-absorbing layer mainly include lead interstitial defects (Pb_i_), iodine interstitial defects (I_i_), and lead–iodine antisite defects (Pb_I_, I_Pb_).^19^ These defects often form deep-level energy defects, which become carrier recombination centers, reducing the device efficiency. In addition, these defects make the perovskite susceptible to erosion by external water or oxygen molecules, which can easily cause perovskite decomposition and affect device stability.^20^ According to the characteristics of these defects, molecular engineering^21−23^ strategies can be used to passivate the perovskite film and effectively reduce carrier recombination. The most common passivation strategy was to use phenethylamine iodide (PEAI) to posttreat the surface of the perovskite film, which can effectively passivate the defects caused by unsaturated coordination of Pb and I vacancies on the perovskite surface,^24,25^ and can improve the surface morphology of the perovskite film, thereby improving the PeSCs efficiency. Some literature reports that the introduction of PEA^+^ can form a PEA_2_PbI_4_ two-dimensional structure on the perovskite surface,^26^ which effectively improves the stability of the PeSCs. In addition, using organic molecules containing −NH_2_ functional groups such as propylammonium chloride (PACl),^27^ hydrazinium iodide (HAI),^28^ and 2-(2-pyridyl)ethylamine (2-PyEA)^29^ to passivate the perovskite surface or bulk phase can also effectively suppress the formation of Pb defects on the perovskite surface. The above passivation strategies mainly solve the problem of surface defect passivation of perovskite thin films, while studies have shown that there are more defects at the bottom interface of perovskite thin films. Zhu et al. studied the morphology, chemical composition, electronic structure, and photophysical properties of the buried interface peeled off by mechanical peeling and confirmed that the buried interface has defects caused by complex phases composed of various nanoscale lead halide microcrystals.^30^ Huang et al. used GIXRD technology to demonstrate that there was a top-down crystallization process during the growth of perovskite films, which makes it easier to form unstable and defective grains at the bottom of the films.^31^ At present, there were few studies on the passivation work of the buried interface, and most of them directly use common upper surface defect passivation materials, which have no significant effect.^32,33^ In addition, due to the polarity mismatch between the passivation material and solvent and the perovskite precursor solvent, the wettability between the perovskite precursor solution and substrate was poor, which can cause problems such as poor coverage of the perovskite film. Therefore, it was necessary to explore better passivation materials for the perovskite buried interface.

It was reported^27,34−37^ that the efficiency and stability of PeSCs can be improved by adding a chloride anion (Cl^–^) to help perovskite grain growth, preferred crystallization orientation, and α-phase stability. Pham et al. proposed that the addition of CsCl/MACl significantly improved the morphology, grain size, and crystallinity of the perovskite films, thereby extending the carrier lifetime. The perovskite layer with CsCl additive achieved the highest PCE of 21.98%.^38^ Park et al. proposed CsCl/MACl dual additives to improve the photovoltaic performance and stability of the PeSCs. MACl was usually used to enhance the phase stability of FAPbI_3_, while CsCl addition reduces the trap density and increases the resistance to charge recombination. The ultimate PCE of dual additives was 23.22%, higher than that of single additives of MACl or CsCl.^39^

Here, Cs_0.05_MA_0.14_FA_0.81_PbI_2.86_Cl_0.14_ was first used as the absorber layer of PeSC, and the doping ratio was adjusted by increasing the concentration of CsCl and decreasing the concentration of MACl. Finally, it was found that the perovskite modulated by this set of ratios of Cs_0.1_MA_0.09_FA_0.81_PbI_2.86_Cl_0.14_ has a better PCE.

In addition, organic ammonium salts were used to passivate the bottom defects of the perovskite film. That is, before depositing the perovskite film, a butane-1,4-diammonium iodide (BDAI_2_) solution was first spin-coated on the substrate as a prepassivation layer. Considering that the three-dimensional structure of the perovskite has been formed in the perovskite precursor solution, it can effectively prevent the diffusion of BDAI_2_ from the bottom into the solution. Therefore, further deposition of perovskite thin films can retain most of the BDAI_2_ at the perovskite buried interface. The study found that this passivation strategy can effectively suppress the defects at the perovskite buried interface, enhance the carrier extraction efficiency at the buried interface, and increase the efficiency of the PeSCs by about 20%.

## 2 Experimental Section

### 2.1 Materials

Patterned FTO-coated glass substrate (7 Ω sq^–1^) was purchased from Ruilong. Cesium chloride (CsCl, 99.99%), lead iodide (PbI_2_, 99.9985%), cesium iodide (CsI, 99.9%), nickel(II) nitrate (99.9985%), ethylenediamine (99%), ethylene glycol (99%), and bathocuproin (BCP, 99.7%) were purchased from Alfa Aesar. Methylammonium chloride (MACl, 99%) was purchased from Thermo Scientific. Formamidinium iodide (FAI, >99.5%) was purchased from Lumtec. Butane-1,4-diammonium iodide (BDAI_2_, 98%), dimethyl sulfoxide (DMSO, 99.5%), *N*,*N*-dimethylformamide (DMF, 99.5%), and fullerene (C_60_, 99.95%) were purchased from Uni-Onward.

### 2.2 Material Fabrication

The NiOx hole transport layer solution was synthesized according to the method described elsewhere.^40^ Dissolve BDAI_2_ powder was dissolved in deionized water solvent to prepare passivation layer solutions with different concentrations of 1, 2, 3, 4, and 5 mg/mL. CsCl/MACl was prepared with different ratios (CsCl/MACl = 0.05/0.14, 0.1/0.09, and 0.15/0.04) and combined them to obtain mixed Cs_0.05_MA_0.14_FA_0.81_PbCl_0.14_I_2.86_, Cs_0.1_MA_0.09_FA_0.81_PbCl_0.14_I_2.86_, and Cs_0.15_MA_0.04_ FA_0.81_PbCl_0.14_I_2.86_ perovskite precursor solutions. Taking the stoichiometric ratio of CsCl/MACl = 0.1/0.09 as an example, the Cs_0.1_MA_0.09_FA_0.81_PbCl_0.14_I_2.86_ perovskite precursor solution was prepared by dissolving CsCl (8.4 mg), MACl (6.1 mg), CsI (13 mg), FAI (140 mg), and PbI_2_ (461 mg) in 1 mL of mixed solvent of DMF and DMSO with a volume ratio of 4:1 at room temperature of 25 °C for about 24 h.

### 2.3 Solar Cell Fabrication

Patterned FTO-coated glass substrates were cleaned thoroughly by sequential ultrasonication for 20 min in an acetone, ethanol, and isopropanol, followed by drying with N_2_ and cleaning in a UV ozone oven for 20 min. The hole transport layer NiOx solution was prepared by spin coating onto the FTO glass substrate at 3000 rpm for 30 s, subsequently to a two-step annealing process at 120 °C for 10 min (hot plate) and 300 °C for 60 min (furnaces). Before spin coating the perovskite film, the passivation material BDAI_2_ solution was spin-coated on the NiOx substrate at 3000 rpm for 30s, then annealed at 150 °C for 10 min as a prepassivation layer, and finally transferred into an N_2_-filled glovebox. CsFAMA mixed-cation perovskite films were deposited on pure NiOx or BDAI_2_-passivated NiOx substrates by a two-step spin-coating method. Spin coating was performed at 1000 and 5000 rpm for 10 and 30 s, respectively, followed by dripping 100 μL of toluene for the remaining 20 s. The as-prepared perovskite films were subsequently annealed on a hot plate at 100 °C for 10 min. In the final step, sequentially, the C_60_ electron transporting layer and BCP electron blocking layer were deposited by thermal evaporating under high vacuum. The PeSC devices were completed by thermally evaporating 100 nm thick Ag to form the top electrode.

### 2.4 Characterization and Measurements

The crystal structure of the perovskite film was characterized using a X’Pert PRO’s MRD X-ray diffractometer (Cu target) produced by PANalytical. The morphology of the perovskite films was taken using a Hitachi’s Regulus 8100 field emission scanning electron microscope (FESEM). The photoluminescence (PL) and absorption spectra were measured using a Hitachi F-7000 fluorescence spectrophotometer and Jasco’s V-770 UV–visible–near infrared (UV/vis/NIR) spectrophotometer, respectively. The Hong-Ming’s MFS-PV-Basic solar simulator was used with a Keithley 2420 sourcemeter to record the current density–voltage (*J*–*V*) curve of the PeSC devices under illumination of air-mass (AM) 1.5G standard sunlight at 100 mW cm^–2^ (calibrated for PV Measurements PVM-894 NREL standard crystalline silicon reference cells). The external quantum efficiency (EQE) was tested using a LiveStrong’s LSQE-R QE measurement system.

## 3 Results and Discussion

**Figure 1:**
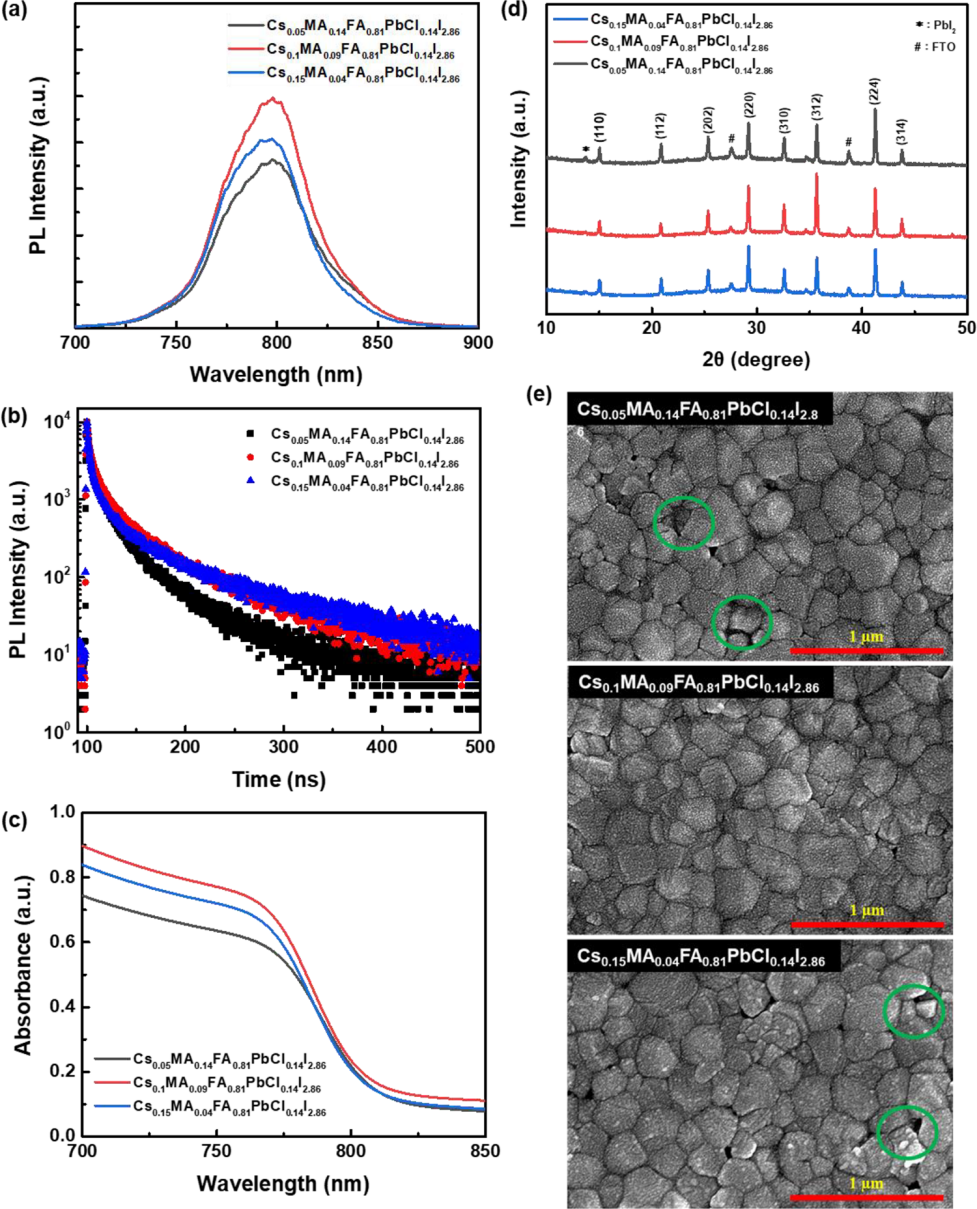
(a) XRD pattern, (b) time-resolved PL, (c) UV–vis absorption, (d) PL Spectra, and (e) top view SEM images of CsFAMA perovskite films with different mixing ratios of dual additive CsCl/MACl.

Figure 1a shows the PL spectra of perovskites doped with different CsCl/MACl ratios of CsCl/MACl. It can be seen that, when the CsCl/MACl ratio was 0.1/0.09, the PL emission peak (the peak was ca. 790 nm) has a better intensity, indicating that Cs_0.1_MA_0.09_FA_0.81_PbCl_0.14_I_2.86_ perovskite has fewer internal traps, better crystallinity, and a slower charge recombination rate. Time-resolved PL (TRPL) was performed to evaluate the recombination behavior of photoexcited carriers in perovskite films (see results in Figure 1b). The TRPL spectra exhibit a much longer lifetime in the Cs_0.1_MA_0.09_FA_0.81_PbCl_0.14_I_2.86_ perovskite film (τ_avg_:7.11 ns) than that of the Cs_0.05_MA_0.14_FA_0.81_PbCl_0.14_I_2.86_ (τ_avg_:4.68 ns) and Cs_0.15_MA_0.04_ FA_0.81_PbCl_0.14_I_2.86_ perovskite films (τ_avg_:5.89 ns). The extended PL lifetime of Cs_0.1_MA_0.09_FA_0.81_PbCl_0.14_I_2.86_ perovskite films can be attributed to higher film quality and fewer structural defects, which effectively suppress trap-assisted recombination and thus suppress nonradiative decay channel. The Cs_0.1_MA_0.09_FA_0.81_PbCl_0.14_I_2.86_ perovskite shows good optical absorption performance in the absorption spectrum (Figure 1c), which was expected to increase Jsc and thus improve the device performance. In addition, it can be observed that the Cs_0.05_MA_0.14_FA_0.81_PbCl_0.14_I_2.86_ perovskite with a CsCl/MACl ratio of 0.05/0.14 exhibits PbI_2_ diffraction peaks (12.7°), as shown in Figure 1d, indicating incomplete conversion to the perovskite film. On the other hand, the Cs_0.1_MA_0.09_FA_0.81_PbCl_0.14_I_2.86_ and Cs_0.15_MA_0.04_ FA_0.81_PbCl_0.14_I_2.86_ perovskites with CsCl/MACl ratios of 0.1/0.09 and 0.15/0.04, respectively, do not have PbI_2_ diffraction peaks. At the same time, the Cs_0.1_MA_0.09_FA_0.81_PbCl_0.14_I_2.86_ perovskite has stronger diffraction peak intensity, indicating a stable α-phase perovskite and improved crystallinity. Figure 1e shows the top view SEM images of perovskites doped with different ratios of CsCl/MACl. As the CsCl ratio increases, it can be seen that the perovskite crystals become larger and denser and the defects were relatively less. However, when CsCl was excessive, the defects increased (as shown by the green circle), resulting in a decrease in the quality of the perovskite film and thus a decrease in the device efficiency. Therefore, an appropriate doping ratio can improve the efficiency of the PeSC.

**Figure 2:**
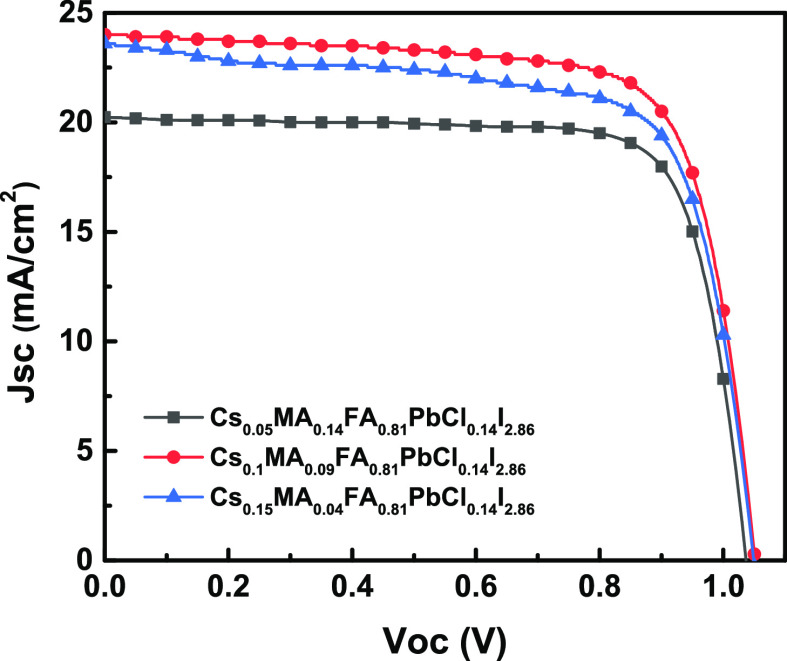
Typical *J*–*V* curves of PeSCs prepared with different mixing ratios of dual additive CsCl/MACl.

Figure 2 shows the *J–V* curves of CsFAMA PeSCs with different mixing ratios of dual additives CsCl/MACl, and the corresponding photovoltaic performance parameters are summarized in Table S1. It can be seen that, as the ratio of CsCl/MACl dual additives increases, the Voc, Jsc, and PCE of PeSCs in each case have an upward trend. Therefore, based on the champion Cs_0.1_MA_0.09_FA_0.81_PbCl_0.14_I_2.86_ PeSC had the best photovoltaic properties, its yields a PCE of 18.6% with a Jsc of 24.0 mA cm^–2^, a Voc of 1.04 V, and an FF of 74.1%. This was attributed to the better crystallinity, fewer pores, and better optical properties of the Cs_0.1_MA_0.09_FA_0.81_PbCl_0.14_I_2.86_ perovskite film, which more effectively reduces nonradiative recombination. However, PeSCs with CsCl/MACl ratios of 0.05/0.14 and 0.15/0.04 have poor photovoltaic performance, mainly due to the appearance of more pores, the δ phase, and PbI_2_, resulting in poor photovoltaic performance.

**Figure 3:**
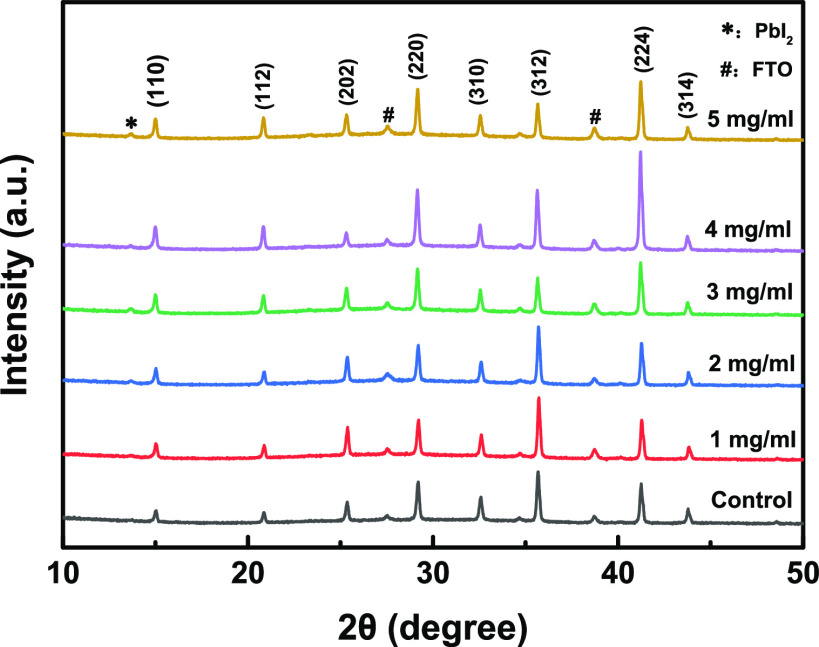
XRD patterns of the CsFAMA perovskite films modified with various BDAI_2_ solution concentrations.

Figure 3 shows the XRD patterns of BDAI_2_-passivated perovskite films at different concentrations. Overall, the XRD diffraction peak intensity of the BDAI_2_-passivated perovskites was better than that of the unpassivated one. Perovskite films based on BDAI_2_ passivation can maintain the 3D perovskite phase, significantly reduce defects, and enhance carrier extraction. However, when the BDAI_2_ concentration was increased to 5 mg/mL, it was shown that there was excess PbI_2_ in the 3D perovskite film at about 12.7° and the intensity of other diffraction peaks was reduced, which may be caused by excess BDA^2+^. Finally, when the BDAI_2_ concentration was 4 mg/mL, the diffraction peaks all have higher intensity, indicating that it has the best crystallinity.

**Figure 4:**
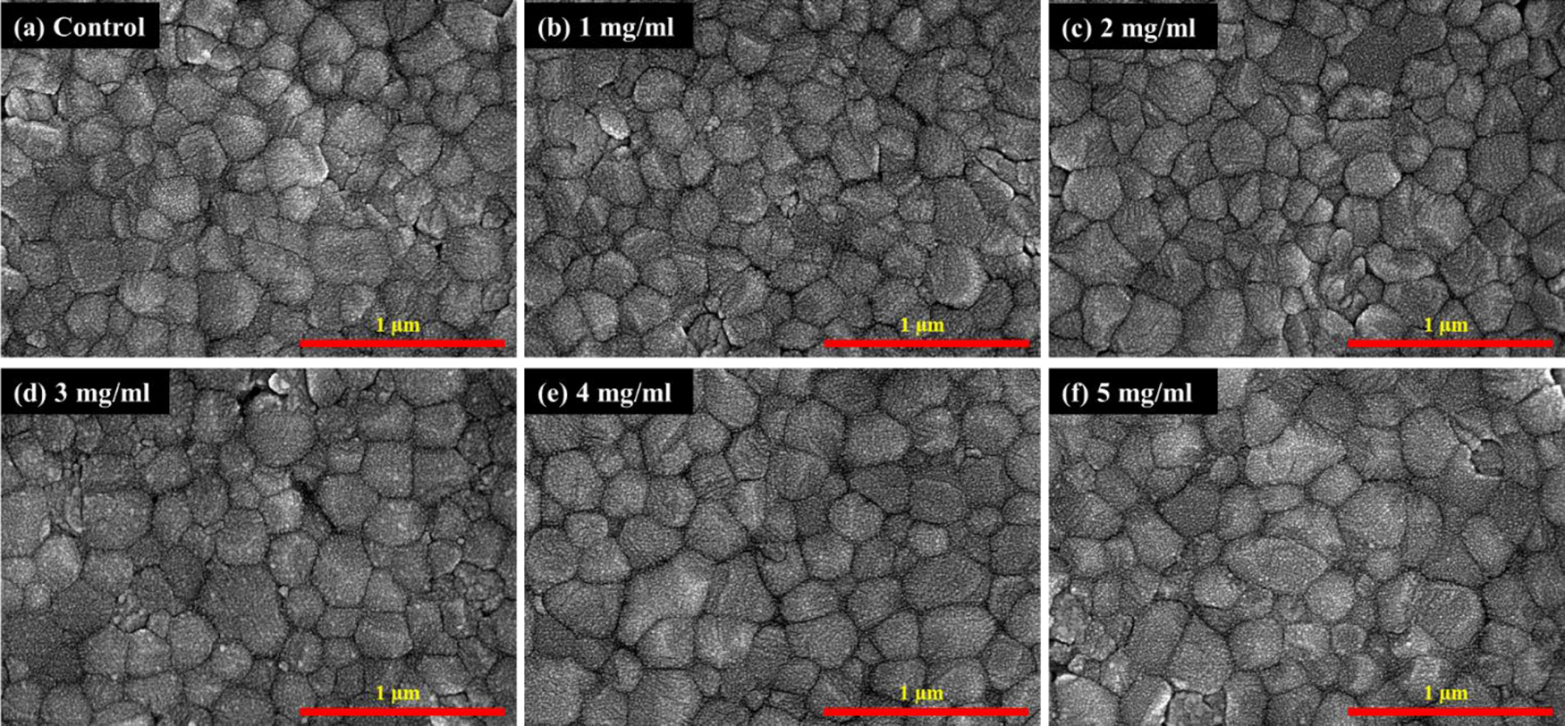
Top view SEM images of the CsFAMA perovskite films modified with various BDAI_2_ solution concentrations.

Figure 4 shows the SEM surface morphology of perovskite films passivated with different concentrations of BDAI_2_. It can be found that the BDAI_2_-passivated perovskite film exhibits increased grain size and fewer holes at a concentration of 4 mg/mL. Since BDAI_2_ serves as a bridging molecule to connect perovskite grains, modifying the grain boundaries will significantly improve the photovoltaic properties and stability of PeSCs. The perovskite film with too much or too little BDAI_2_ concentration was not very crystalline and showed a poor surface morphology with pinholes, which was due to the destruction of the 3D spatial structure of the perovskite.

**Figure 5:**
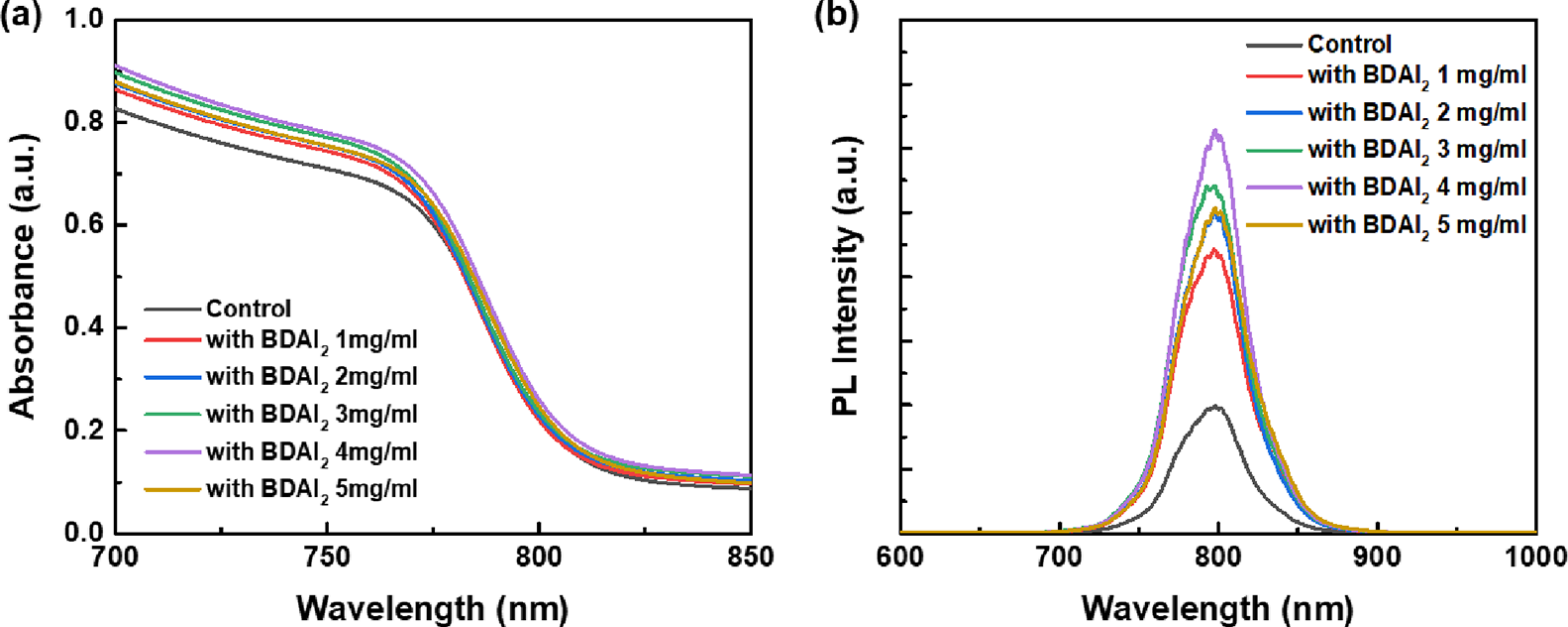
(a) Absorption and (b) PL spectra of CsFAMA perovskite films modified with various BDAI_2_ solution concentrations.

To further analyze the perovskite films, absorption spectroscopy measurements were performed. As shown in Figure 5a, adding a BDAI_2_-passivated layer can enhance the absorption intensity, which can be attributed to the better crystallinity of the perovskite film. When the BDAI_2_ concentration was increased to 4 mg/mL, it shows better light absorption, so the interface passivation of the perovskite film can be used to obtain better crystallinity and fewer holes and defects, thereby improving the Jsc of PeSC. Figure 5b shows the PL spectra of perovskite films passivated by BDAI_2_ solutions with different concentrations, which was consistent with the absorption spectrum analysis. When the concentration of BDAI_2_ increases, the peak intensity of its PL will also increase. There was the strongest PL peak at 4 mg/mL, which was located near 790 nm, indicating that the perovskite film has good crystallinity.

**Figure 6:**
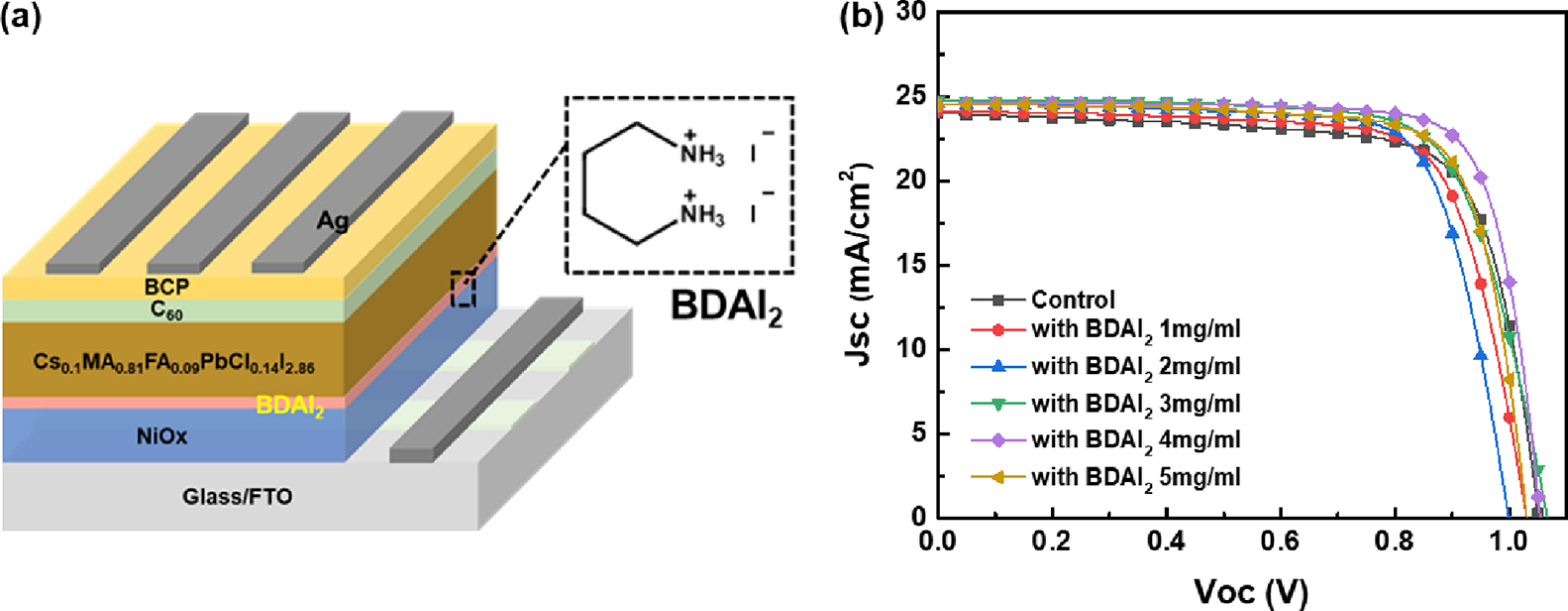
(a) Schematic illustration of the PeSC device with a BDAI_2_-passivated layer configuration and (b) *J*–*V* curves of PeSCs modified with various BDAI_2_ solution concentrations.

To explore the effect of perovskite buried interface passivation on the performance of PeSC devices, we constructed an inverted planar solar cell with the structure ITO/NiOx/BDAI_2_/CSFAMA perovskite/C_60_/BCP/Ag. Among them, ITO was the anode (transparent electrode), Ag was the cathode (back electrode), NiOx was the hole transport layer, C_60_ was the electron transport layer, BCP was the hole blocking layer, and perovskite CSFAMA was the light-absorbing layer, as shown in Figure 6a. The photovoltaic performance parameter results of the PeSCs were shown in Figure 6b, Figure S1, and Table S2. From Figure S1, it was found that Jsc increased significantly after BDAI_2_ treatment, its PCE was higher than that of original PeSC, and the best photovoltaic performance was obtained when the solution concentration was 4 mg/mL. Figure 6b and Table S2 show that, compared with the original PeSC, after passivating the buried interface with 3 mg/mL BDAI_2_, the Voc, Jsc, FF, and PCE parameters of the PeSC have been significantly improved, which were 1.05 V, 24.7 mA cm^–2^, 73.7%, and 19.2%, respectively. Further increasing the concentration of BDAI_2_ passivation material to 4 mg/mL, the Voc, Jsc, FF, and PCE parameters of the PeSC continue to increase to 1.04 V, 24.5 mA cm^–2^, 79.9%, and 20.5%. The significant increase in the device performance of PeSCs based on BDAI_2_ passivation, especially the Jsc and FF parameters, indicates that the degree of carrier recombination inside the device was significantly reduced. When the concentration of the passivator BDAI_2_ was increased to 5 mg/mL, the Voc, Jsc, FF, and PCE parameters of the PeSC were 1.02 V, 24.5 mA cm^–2^, 77.0%, and 19.3%, respectively. Compared with 4 mg/mL, the device parameters have declined slightly, which shows that excessive BDA^2+^ was distributed at the perovskite buried interface, which will increase the resistance of the buried interface and have a negative effect on PeSC performance.

**Figure 7:**
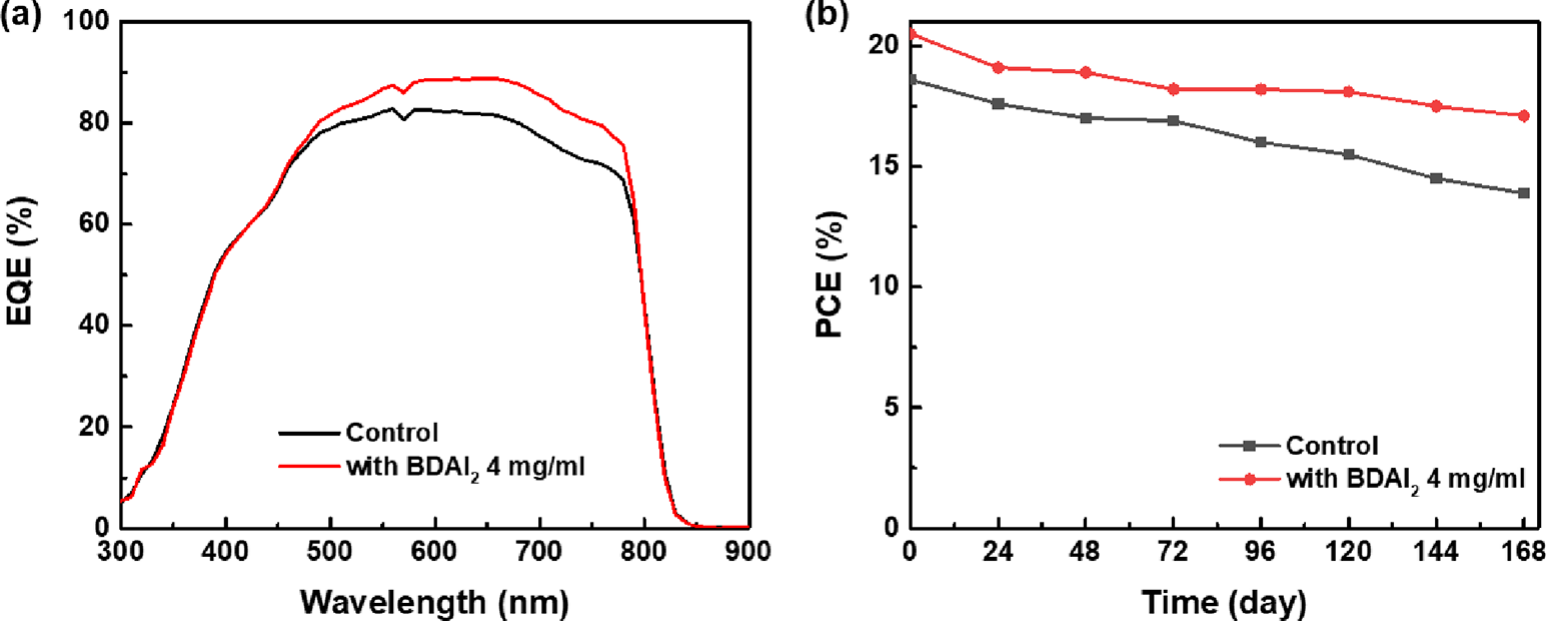
(a) EQE spectra and (b) PCE stability evolution (N_2_-filled glovebox) of PeSCs with and without BDAI_2_ passivation.

Figure 7a shows the EQE of the best PeSCs device before and after BDAI_2_ passivation. There was no significant change in the shape of the EQE curve of the PeSC before and after passivation, but the EQE curve of the BDAI_2_-passivated PeSC was basically higher than that of the original PeSC in the entire wavelength range; therefore, its device exhibits a higher Jsc. To further investigate the impact of BDAI_2_ passivation on the performance of PeSCs devices, device stability testing was conducted. As shown in Figure 7b, after the device was stored for 168 h, the efficiency of PeSCs based on original and BDAI_2_ passivation decreased by 26 and 17%, respectively. It can be seen that the stability of PeSc was significantly enhanced after passivation with BDAI_2_.

## 4 Conclusions

In conclusion, the perovskite was first modulated with different doping ratios of CsCl and MACl. The perovskite with Cs_0.1_MA_0.09_FA_0.81_PbCl_0.14_I_2.86_ has better absorbance and crystallinity, achieving an 18.6% PeSC performance. To further explore the performance improvement of PeSCs, we proposed an effective strategy to control the growth of the perovskite film by depositing BDAI_2_ between NiOx and the perovskite layer in the p-i-n inverted structure to passivate the buried interface defects of the perovskite layer. Inserting the BDAI_2_ passivation layer does not affect the crystal structure and morphology of the perovskite layer and improves the optoelectronic properties of the perovskite film. In addition, the use of BDAI_2_ can simultaneously suppress the nonradiative energy loss at the top and bottom interfaces of the perovskite. As a result, the Jsc of 24.0 mA cm^–2^ was improved to 24.5 mA cm^–2^, the FF of 74.1% was greatly improved to 79.9%, and the optimal PCE can reach 20.5%.

## Supporting Information Available

Statistical data of Voc, Jsc, FF, and PCE for PeSCs modified with various BDAI_2_ solution concentrations; performance parameters of PeSCs prepared with different mixing ratio of dual additive CsCl/MACl; and performance parameters of PeSCs modified with various BDAI_2_ solution concentrations (PDF)

## References

[ref1] NREL. Interactive best research-cell efficiency chart [EB/OL]. [2024-01–31]. https://www.nrel.gov/pv/interactive-cell-efficiency.html.

[ref2] Zhao Y.; Ma F.; Qu Z.; Yu S.; Shen T.; Deng H. X.; Chu X.; Peng X.; Yuan Y.; Zhang X.; You J. Inactive (PbI_2_)_2_RbCl stabilizes perovskite films for efficient solar cells. Science 2022, 377 (6605), 531–534. 10.1126/science.abp8873. 35901131

[ref3] Yoo J. J.; Seo G.; Chua M. R.; Park T. G.; Lu Y.; Rotermund F.; Kim Y. K.; Moon C. S.; Jeon N. J.; Correa-Baena J. P.; Bulović V.; Shin S. S.; Bawendi M. G.; Seo J. Efficient perovskite solar cells via improved carrier management. Nature 2021, 590, 587–593. 10.1038/s41586-021-03285-w. 33627807

[ref4] Wojciechowski K.; Forgács D. Commercial applications of indoor photovoltaics based on flexible perovskite solar cells. ACS Energy Lett. 2022, 7 (10), 3729–3733. 10.1021/acsenergylett.2c01976.

[ref5] Zhang X.; Qiu W.; Song W.; Hawash Z.; Wang Y.; Pradhan B.; Zhang Y.; Naumenko D.; Amenitsch H.; Moons E.; Merckx T.; Aguirre A.; Abdulraheem Y.; Aernouts T.; Zhan Y.; Kuang Y.; Hofkens J.; Poortmans J. An integrated bulk and surface modification strategy for gas-quenched inverted perovskite solar cells with efficiencies exceeding 22%. Sol. RRL 2022, 6 (6), 2200053 10.1002/solr.202200053.

[ref6] Li Z.; Li B.; Wu X.; Sheppard S. A.; Zhang S.; Gao D.; Long N. J.; Zhu Z. Organometallic-functionalized interfaces for highly efficient inverted perovskite solar cells. Science 2022, 376 (6591), 416–420. 10.1126/science.abm8566. 35446656

[ref7] Jiang Q.; Tong J.; Xian Y.; Kerner R. A.; Dunfield S. P.; Xiao C.; Scheidt R. A.; Kuciauskas D.; Wang X.; Hautzinger M. P.; Tirawat R.; Beard M. C.; Fenning D. P.; Berry J. J.; Larson B. W.; Yan Y.; Zhu K. Surface reaction for efficient and stable inverted perovskite solar cells. Nature 2022, 611 (7935), 278–283. 10.1038/s41586-022-05268-x. 36049505

[ref8] Mushtaq A.; Pradhan B.; Kushavah D.; Zhang Y.; Naumenko D.; Amenitsch H.; Hofkens J.; Pal S. K. Femtosecond induced third-order optical nonlinearity in quasi 2D Ruddlesden–Popper perovskite film deciphered using Z-scan. Mater. Adv. 2022, 3 (22), 8211–8219. 10.1039/D2MA00724J.

[ref9] Cai B.; Ma Y.; Yang B.; Liu Y.; Xia J.; Chen X.; Li Z.; Ju M. G. A new descriptor for complicated effects of electronic density of states on ion migration. Adv. Funct. Mater. 2023, 33 (29), 2300445 10.1002/adfm.202300445.

[ref10] Saliba M. Polyelemental, multicomponent perovskite semiconductor libraries through combinatorial screening. Adv. Energy Mater. 2019, 9 (25), 1803754 10.1002/aenm.201803754.

[ref11] Jena A. K.; Kulkarni A.; Miyasaka T. Halide perovskite photovoltaics: background, status, and future prospects. Chem. Rev. 2019, 119 (5), 3036–3103. 10.1021/acs.chemrev.8b00539. 30821144

[ref12] Jian W.; Jia R.; Zhang H. X.; Bai F. Q. Arranging strategies for A-site cations: impact on the stability and carrier migration of hybrid perovskite materials. Inorg. Chem. Front. 2020, 7 (8), 1741–1749. 10.1039/D0QI00102C.

[ref13] Saliba M.; Matsui T.; Seo J.-Y.; Domanski K.; Correa-Baena J.-P.; Nazeeruddin M. K.; Zakeeruddin S. M.; Tress W.; Abate A.; Hagfeldt A.; Grätzel M. Cesium-containing triple cation perovskite solar cells: improved stability, reproducibility and high efficiency. Energy Environ. Sci. 2016, 9 (6), 1989–1997. 10.1039/C5EE03874J. 27478500 PMC4936376

[ref14] Mahboubi Soufiani A.; Yang Z.; Young T.; Miyata A.; Surrente A.; Pascoe A.; Galkowski K.; Abdi-Jalebi M.; Brenes R.; Urban J.; Zhang N.; Bulović V.; Portugall O.; Cheng Y.-B.; Nicholas R. J.; Ho-Baillie A.; Green M. A.; Plochocka P.; Stranks S. D. Impact of microstructure on the electron–hole interaction in lead halide perovskites. Energy Environ. Sci. 2017, 10 (6), 1358–1366. 10.1039/C7EE00685C.

[ref15] Wu X.; Jiang Y.; Chen C.; Guo J.; Kong X.; Feng Y.; Wu S.; Gao X.; Lu X.; Wang Q.; Zhou G.; Chen Y.; Liu J. M.; Kempa K.; Gao J. Stable triple cation perovskite precursor for highly efficient perovskite solar cells enabled by interaction with 18C6 stabilizer. Adv. Funct. Mater. 2020, 30 (6), 1908613 10.1002/adfm.201908613.

[ref16] Lin X.; Cui D.; Luo X.; Zhang C.; Han Q.; Wang Y.; Han L. Efficiency progress of inverted perovskite solar cells. Energy Environ. Sci. 2020, 13 (11), 3823–3847. 10.1039/D0EE02017F.

[ref17] Chen P.; Bai Y.; Wang L. Minimizing voltage losses in perovskite solar cells. Small Struct. 2021, 2 (1), 2000050 10.1002/sstr.202000050.

[ref18] Fu L.; Li H.; Wang L.; Yin R.; Li B.; Yin L. Defect passivation strategies in perovskites for an enhanced photovoltaic performance. Energy Environ. Sci. 2020, 13 (11), 4017–4056. 10.1039/D0EE01767A.

[ref19] Buin A.; Pietsch P.; Xu J.; Voznyy O.; Ip A. H.; Comin R.; Sargent E. H. Materials processing routes to trap-free halide perovskites. Nano Lett. 2014, 14 (11), 6281–6286. 10.1021/nl502612m. 25296282

[ref20] Ono L. K.; Liu S.; Qi Y. Reducing detrimental defects for high-performance metal halide perovskite solar cells. Angew. Chem., Int. Ed. 2020, 59 (17), 6676–6698. 10.1002/anie.201905521. PMC7187320 31369195

[ref21] Isikgor F. H.; Zhumagali S.; T. Merino L. V.; De Bastiani M.; McCulloch I.; De Wolf S. Molecular engineering of contact interfaces for high-performance perovskite solar cells. Nat. Rev. Mater. 2023, 8, 89–108. 10.1038/s41578-022-00503-3.

[ref22] Chavan R. D.; Bończak B.; Kruszyńska J.; Mahapatra A.; Ans M.; Nawrocki J.; Nikiforow K.; Yadav P.; Paczesny J.; Sadegh F.; Unal M.; Akin S.; Prochowicz D. Molecular engineering of azahomofullerene-based electron transporting materials for efficient and stable perovskite solar cells. Chem. Mater. 2023, 35 (19), 8309–8320. 10.1021/acs.chemmater.3c01995.

[ref23] Sun J.; Ma K.; Lin Z. Y.; Tang Y.; Varadharajan D.; Chen A. X.; Atapattu H. R.; Lee Y. H.; Chen K.; Boudouris B. W.; Graham K. R.; Lipomi D. J.; Mei J.; Savoie B. M.; Dou L. Tailoring molecular-scale contact at the perovskite/polymeric hole-transporting material interface for efficient solar cells. Adv. Mater. 2023, 35 (26), 2300647 10.1002/adma.202300647. 36942854

[ref24] Jiang Q.; Zhao Y.; Zhang X.; Yang X.; Chen Y.; Chu Z.; Ye Q.; Li X.; Yin Z.; You J. Surface passivation of perovskite film for efficient solar cells. Nat. Photonics 2019, 13 (7), 460–466. 10.1038/s41566-019-0398-2.

[ref25] Zhuang J.; Mao P.; Luan Y.; Yi X.; Tu Z.; Zhang Y.; Yi Y.; Wei Y.; Chen N.; Lin T.; Wang F.; Li C.; Wang J. Interfacial passivation for perovskite solar cells: the effects of the functional group in phenethylammonium iodide. ACS Energy Lett. 2019, 4 (12), 2913–2921. 10.1021/acsenergylett.9b02375.

[ref26] Wang T.; Fu Y.; Jin L.; Deng S.; Pan D.; Dong L.; Jin S.; Huang L. Phenethylammonium functionalization enhances near-surface carrier diffusion in hybrid perovskites. J. Am. Chem. Soc. 2020, 142 (38), 16254–16264. 10.1021/jacs.0c04377. 32845129

[ref27] Zhang Y.; Li Y.; Zhang L.; Hu H.; Tang Z.; Xu B.; Park N. G. Propylammonium chloride additive for efficient and stable FAPbI3 perovskite solar cells. Adv. Energy Mater. 2021, 11 (47), 2102538 10.1002/aenm.202102538.

[ref28] Zhang K.; Späth A.; Almora O.; Le Corre V. M.; Wortmann J.; Zhang J.; Xie Z.; Barabash A.; Hammer M. S.; Heumüller T.; Min J.; Fink R.; Lüer L.; Li N.; Brabec C. J. Suppressing nonradiative recombination in lead-tin perovskite solar cells through bulk and surface passivation to reduce open circuit voltage losses. ACS Energy Lett. 2022, 7 (10), 3235–3243. 10.1021/acsenergylett.2c01605.

[ref29] Li G.; Song J.; Wu J.; Song Z.; Wang X.; Sun W.; Fan L.; Lin J.; Huang M.; Lan Z.; Gao P. Efficient and stable 2D@3D/2D perovskite solar cells based on dual optimization of grain boundary and interface. ACS Energy Lett. 2021, 6 (10), 3614–3623. 10.1021/acsenergylett.1c01649.

[ref30] Yang X.; Luo D.; Xiang Y.; Zhao L.; Anaya M.; Shen Y.; Wu J.; Yang W.; Chiang Y.; Tu Y.; Su R.; Hu Q.; Yu H.; Shao G.; Huang W.; Russell T. P.; Gong Q.; Stranks S. D.; Zhang W.; Zhu R. Buried interfaces in halide perovskite photovoltaics. Adv. Mater. 2021, 33 (7), 2006435 10.1002/adma.202006435. 33393159

[ref31] Chen S.; Xiao X.; Chen B.; Kelly L. L.; Zhao J.; Lin Y.; Toney M. F.; Huang J. Crystallization in one-step solution deposition of perovskite films: upward or downward?. Sci. Adv. 2021, 7 (4), eabb2412 10.1126/sciadv.abb2412. 33523938 PMC10670903

[ref32] Chen B.; Chen H.; Hou Y.; Xu J.; Teale S.; Bertens K.; Chen H.; Proppe A.; Zhou Q.; Yu D.; Xu K.; Vafaie M.; Liu Y.; Dong Y.; Jung E. H.; Zheng C.; Zhu T.; Ning Z.; Sargent E. H. Passivation of the buried interface via preferential crystallization of 2D perovskite on metal oxide transport layers. Adv. Mater. 2021, 33 (41), 2103394 10.1002/adma.202103394. 34425038

[ref33] Yin X.; Zhai J.; Du P.; Li N.; Song L.; Xiong J.; Ko F. 3D NiO nanowall hole-transporting layer for the passivation of interfacial contact in inverted perovskite solar cells[J]. ChemSusChem 2020, 13 (5), 1006–1012. 10.1002/cssc.201903025. 31898849

[ref34] Kim M.; Lee T. K.; Choi I. W.; Choi H. W.; Jo Y.; Lee J.; Kim G. H.; Kwak S. K.; Kim D. S. Effects of cation size and concentration of cationic chlorides on the properties of formamidinium lead iodide based perovskite solar cells. Sustainable Energy Fuels 2020, 4, 3753–3763. 10.1039/D0SE00382D.

[ref35] Chavan R. D.; Prochowicz D.; Yadav P.; Tavakoli M. M.; Nimbalkar A.; Bhoite S. P.; Hong C. K. Effect of CsCl additive on the morphological and optoelectronic properties of formamidinium lead iodide perovskite. Sol. RRL 2019, 3, 1900294 10.1002/solr.201900294.

[ref36] Lyu M.; Park N. G. Effect of additives AX (A = FA, MA, Cs, Rb, NH_4_, X = Cl, Br, I) in FAPbI_3_ on photovoltaic parameters of perovskite solar cells. Sol. RRL 2020, 4, 2000331 10.1002/solr.202000331.

[ref37] Kim M.; Kim G. H.; Lee T. K.; Choi I. W.; Choi H. W.; Jo Y.; Yoon Y. J.; Kim J. W.; Lee J.; Huh D.; Lee H.; Kwak S. K.; Kim J. Y.; Kim D. S. Methylammonium chloride induces intermediate phase stabilization for efficient perovskite solar cells. Joule 2019, 3, 2179–2192. 10.1016/j.joule.2019.06.014.

[ref38] Pham H. T.; Yin Y.; Andersson G.; Weber K. J.; Duong T.; Wong-Leung J. Unraveling the influence of CsCl/MACl on the formation of nanotwins, stacking faults and cubic supercell structure in FA-based perovskite solar cells. Nano Energy 2021, 87, 106226 10.1016/j.nanoen.2021.106226.

[ref39] Yang S.; Park N.-G. Dual additive for simultaneous improvement of photovoltaic performance and stability of perovskite solar cell. Adv. Funct. Mater. 2021, 30 (20), 2100396 10.1002/adfm.202100396.

[ref40] Tien C. H.; Lai H. Y.; Chen L. C. Methylammonium halide salt interfacial modification of perovskite quantum dots/triple-cation perovskites enable efficient solar cells. Sci. Rep. 2023, 13, 5387 10.1038/s41598-023-32697-z. 37012304 PMC10070349

