# Supplementary material for: Buried Interface Passivation Using Organic Ammonium Salts for Efficient Inverted CsMAFA Perovskite Solar Cell Performance
Source: ACS Omega. 2024 May 16;9(21):23033–9. doi: 10.1021/acsomega.4c02656 (PMC11137706; doi:10.1021/acsomega.4c02656)
Supplement: Supplementary file 1 — ao4c02656_si_001.pdf [file ao4c02656_si_001.pdf]

Supplementary Information

Buried interface passivation using organic ammonium salts for efficient inverted CsMAFA perovskite solar cells performance

Ching-Ho Tien[1,2,*], Wei-Shuo Lai[3], and Lung-Chien Chen[3,*]

[1]Department of Electronic Engineering, Ming Chi University of Technology, No. 84, Gungjuan Rd., New Taipei City 24301, Taiwan; chtien@mail.mcut.edu.tw (C.-H.T.)

[2]Organic Electronics Research Center, Ming Chi University of Technology, No. 84, Gungjuan Rd., New Taipei City 24301, Taiwan.

[3]Department of Electro-Optical Engineering, National Taipei University of Technology, No. 1, Sec. 3, Chung-Hsiao E. Rd., Taipei 10608, Taiwan; willson880221@gmail.com (W.-S.L.)

Corresponding Author:

*E-mail: chtien@mail.mcut.edu.tw and ocean@ntut.edu.tw

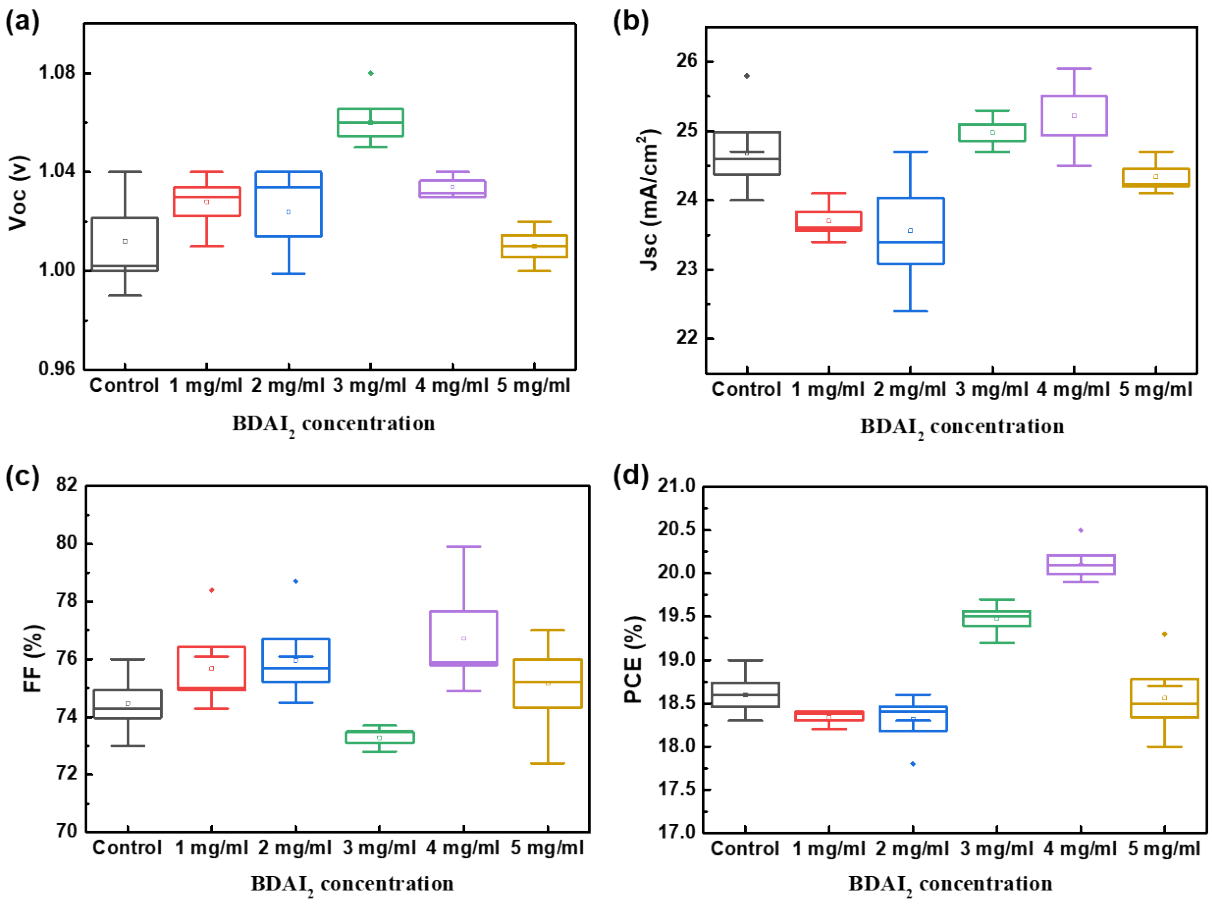

Figure S1. Statistical data of (a) Voc, (b) Jsc, (c) FF, and (d) PCE for PeSCs modified with various $BDAI_2$ solution concentrations.

Table S1. Performance parameters of PeSCs prepared with different mixing ratio of dual additive CsCl/MACl.

Sample	Jsc (mA cm^{-2})	Voc (V)	FF (%)	PCE (%)
$Cs_{0.05}MA_{0.14}FA_{0.81}PbCl_{0.14}I_{2.86}$	20.2	1.02	78.7	16.3
$Cs_{0.1}MA_{0.09}FA_{0.81}PbCl_{0.14}I_{2.86}$	24.0	1.04	74.1	18.6
$Cs_{0.15}MA_{0.04}FA_{0.81}PbCl_{0.14}I_{2.86}$	23.0	1.03	72.0	17.6

Table S2. Performance parameters of PeSCs modified with various $BDAI_2$ solution concentrations.

Sample	Jsc (mA cm^{-2})	Voc (V)	FF (%)	PCE (%)
Control	24.0	1.04	74.1	18.6
$BDAI_2$ 1 mg/ml	24.1	1.02	74.3	18.4
$BDAI_2$ 2 mg/ml	24.6	1.00	74.8	18.3
$BDAI_2$ 3 mg/ml	24.7	1.05	73.7	19.2
$BDAI_2$ 4 mg/ml	24.5	1.04	79.9	20.5
$BDAI_2$ 5 mg/ml	24.5	1.02	77.0	19.3
